# Anti-cancer effects of aloe-emodin: a systematic review

**Published:** 2017-09-07

**Authors:** Brian Sanders, Anna M. Ray, Sharon Goldberg, Tyler Clark, H. Reginald McDaniel, Steven E. Atlas, Ashar Farooqi, Janet Konefal, Lucas C. Lages, Johanna Lopez, Ammar Rasul, Eduard Tiozzo, Judi M. Woolger, John E. Lewis

**Affiliations:** ^1^Department of Medicine, University of Miami Miller School of Medicine, Miami, Florida, United States; ^2^Glow Health, PA, Bay Harbor Islands, Florida, United States; ^3^Wellness Quest, LLC, Grand Prairie, Texas, United States; ^4^Department of Psychiatry & Behavioral Sciences, University of Miami Miller School of Medicine, Miami, Florida, United States; ^5^Department of Family Medicine, University of Miami Miller School of Medicine, Miami, Florida, United States

**Keywords:** aloe vera, anthraquinones, anti-cancer properties, tumor biochemistry, immune signaling, *in vitro*molecular pharmacology

## Abstract

**Background:**

Anthraquinones are a possible treatment option for oncological patients due to their anti-cancer properties. Cancer patients often exhaust a plethora of resources that ultimately fail to provide fully curative measures. Alternative treatments are subsequently sought in the hope of finding a therapeutic remedy. Po¬tential regimens include aloe-emodin and its related derivatives. This review therefore summarizes the effects of aloe-emodin and other aloe components in light of their anti-proliferative and anti-carcinogenic properties.

**Methods:**

A systematic search was performed in PubMed for aloe-emodin and cancer in humans. Sixty abstracts of *in vitro*studies were selected and reviewed with subsequent screening of the full text. Thirty-eight articles were summarized.

**Results:**

Aloe-emodin possesses multiple anti-proliferative and anti-carcinogenic properties in a host of human cancer cell lines, with often multiple vital pathways affected by the same molecule. The most notable effects include inhibition of cell proliferation, migration, and invasion; cycle arrest; induction of cell death; mitochondrial membrane and redox perturbations; and modulation of immune signaling. The effects of aloe-emodin are not ubiquitous across all cell lines but depend on cell type.

**Conclusions:**

On the basis of this systematic review, the multiple consistent effects of aloe-emodin in hu¬man-derived cancer cell lines suggest that aloe-emodin is a potential anti-cancer agent that acts on cancer cells in a pleiotropic manner.

**Relevance for patients:**

Cancer patients often utilize alternative therapies as a result of suboptimal efficacy of conventional treatments. Aloe-emodin might become a therapeutic option for cancer patients if the basic research is confirmed in clinical trials.

## 1. Introduction

Cancer incidence and prevalence are increasing in the United States, placing a heavy burden on affected individuals and caregivers [1]. Conventional cancer treatment, consisting of surgery, chemotherapy, and/or radiation, is commonly asso¬ciated with significant morbidity, and cure rates for many can¬cers are suboptimal [2]. It is for those reasons, presumably, that cancer patients show great interest in complementary therapies, such as nutraceuticals, both for symptom reduction and in the post-treatment survivorship period.

**Figure 1:**
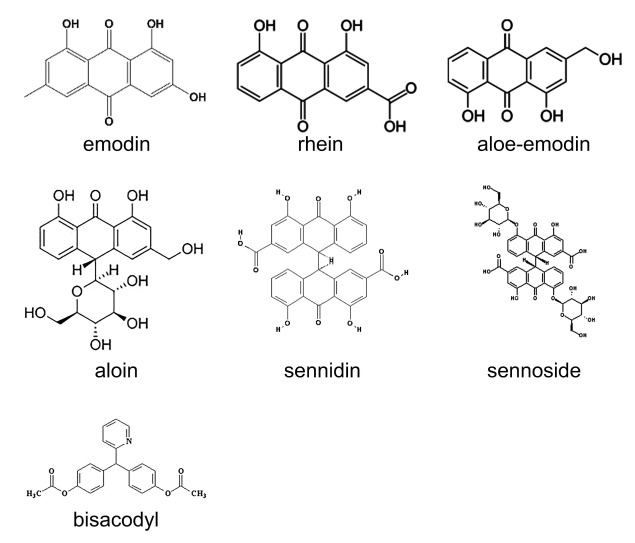
Chemical structure of aloe-emodin and structurally related anthraquinones addressed in this paper.

Supplementing the diet with nutraceuticals containing con-centrated levels of bioactive nutrients, as opposed to obtaining those nutrients solely from food, can be beneficial. Certain anthraquinones, such as aloe-emodin and rhein (Figure 1), are phytochemicals that can be used to restore compromised health [3]. Aloe-emodin is one of many bioactive anthraqui-none components of aloe vera *(Aloe barbadensis miller),*a perennial cactus-like plant found in tropical climates world¬wide. Aloe has been used as a traditional remedy in many cul¬tures for centuries, and it continues to be extremely popular among both cancer and non-cancer patients [4]. Aloe-emodin possesses numerous beneficial biochemical properties. The compound has been used as an anti-inflammatory agent, an immunomodulator, and mediator of wound healing [5]. The most notable effect is that of an antineoplastic agent.

Despite an impressive array of *in vitro*antineoplastic effects, a paucity of clinical research exists on aloe-emodin. Further¬more, aloe vera is used worldwide in an unregulated manner, particularly among cancer patients. Most research has focused on determining the molecular mechanism of current treatments as opposed to creating new therapies. For these reasons, we aim to review the molecular mechanisms of mainly aloe-em-odin and structurally related anthraquinones in cancer cells to highlight its oncopharmacological properties. The chemical structure of aloe-emodin has been previously characterized by others [6], but no one has systematically reviewed the an¬ti-cancer effects of aloe-emodin or structurally related anthra-quinones in human-derived cancer cell lines.

## 2. Methods

A systematic search for articles was performed using Pub-Med. Articles published in English between 1989 and 2015 with full text available were searched using the terms "aloe-emodin," "cancer," "aloe vera," and "humans." Inclusion cri¬teria were: (a) *in vitro*study using human-derived cancer cell lines; (b) use of aloe-emodin or structurally related anthraqui-none as therapeutic agent; and (c) evaluation of the therapeutic agent on at least one marker of tumor cell proliferation. Two independent reviewers evaluated the articles for inclusion in this review.

## 3. Results

**Figure 2.**
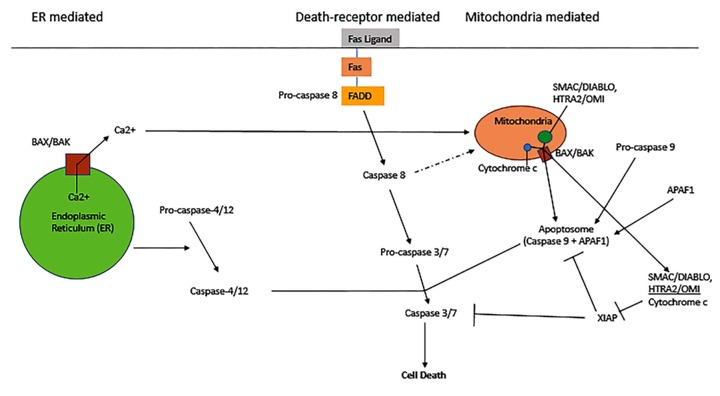
Apoptotic pathways and their regulation. Adapted from: http://www.nature.com/nrm/journal/v6/n4/images/nrm1618-i2.jpg

**Figure 3.**
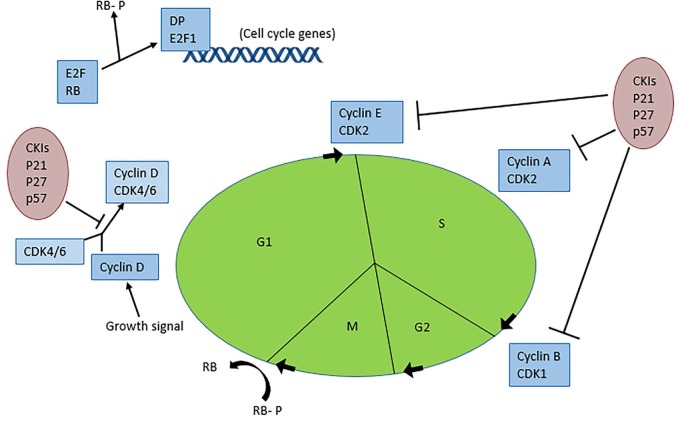
The cell cycle and its regulating factors. Adapted from: http://www.nature.com/nrn/journal/v8/n5/box/nrn2124_BX1.html.

The search resulted in 183 articles, of which 60 were identi¬fied from title and abstract prior to screening with the inclu¬sion criteria. Full text screening identified the articles that met inclusion criteria. Ultimately, 38 *in vitro*studies of human tu¬mor cells were included in this review; the results of which are listed by origin of cancer and/or cell line (Table 1). The main findings are addressed in the text, while the specific pro¬tein/enzyme changes are described in (Table 1),

In summary, aloe-emodin exhibits an array of anti-tumor effects in various cancer cell lines, including induction of apoptosis, cell cycle arrest, modulation of immune signaling, and cell mobility alterations. Aloe-emodin reduces cancer cell viability through extrinsic (TNF-a and FASL) and intrinsic (cytochrome c/caspase 9) apoptosis pathways, which coincide with deleterious effects on mitochondrial membrane permea-bility and/or oxidative stress via exacerbated ROS production. The apoptotic pathways are illustrated in (Figure 2) Aloe-e-modin further causes cell cycle arrest through cyclin and cy-clin-dependent kinase downregulation. The cell cycle path¬ways and molecular regulators are depicted in (Figure 3) Al-oe-emodin also decreased transcription factor activity and al¬tered transcriptional expression and/or protein levels of nu-merous cell signaling proteins important in proliferation and metabolism. In certain cancer cell lines, aloe-emodin induced immune signaling by upregulating, activating, and/or releasing interleukins, GM-CSF, NF-KB, and growth factors. Finally, by altering cell migration, invasion, and adhesion, aloe-emodin negatively affected tumor cell outgrowth propensity.

## 3.1. Bladder cancer

In bladder cancer cells (T24), aloe-emodin induced time-and dose-dependent apoptosis [7]. The cell death induction was accompanied by perturbation of mitochondrial membrane potential and reduced levels of cyclin-dependent kinase (CDK) 1, cyclin B1, and BCL-2 after treatment with aloe-emodin.

## 3.2. Cervical cancer

Cervical cancer cells (HeLa) were treated with aloe-emodin, which caused cell cycle arrest in the G2/M phase. The cells showed a decrease in cyclin A and CDK2, which reduces the cell's ability to proliferate, and suppression of protein kinase Ca (PKCa) and c-MYC, signifying that proliferation and dif¬ferentiation were suppressed [8]. Increases in cyclin B1, CDK1, and alkaline phosphatase (ALP) activity were observed along with inhibition of proliferating cell nuclear antigen (PCNA), showing decreased proliferation.

## 3.3. Colon cancer

It has been previously shown that 1,8-dihydroxyanthra-quinone (DHA) laxatives are associated with colon cancer development [9]. SW480 carcinoma cells, VACO235 adenoma cells, and normal colonic epithelium were treated with various DHA laxatives to determine their effects. SW480 carcinoma cells showed a dose-dependent increase in urokinase secretion (an enzyme that digests extracellular matrix, which could in¬crease tumor cell migration and metastasis, but also causes cell lysis) that caused a reduction in cell numbers by DHA-agly-cones. Rhein and aloe-emodin (types of DHA laxatives) in¬creased BrdU (5-bromo-2'-desoxyuridine; a marker of cell proliferation) by 37% and 50%, respectively. In contrast, pre-malignant VACO235 adenoma cells did not show an increase in urokinase secretion by sennidine A/B and aloe-emodin. However, cell growth and DNA synthesis increased as reflect-ed by elevated BrdU staining. DHA laxatives had no effect on the normal colorectal epithelium [9]. The anti-proliferative effect of aloe-emodin in WiDr cells (colon cancer cell type) was shown by suppression of phorbol-12-myristyl-13-acetate (PMA), which induces tumor migration and invasion [10]. Aloe-emodin downregulated messenger RNA expression and promoter/ gelatinolytic activity of matrix metalloproteinase (MMP)-2/9 and decreased Ras homologue gene family mem-ber B (RHOB) expression. Nuclear translocation of and DNA binding by NF-KB were suppressed along with vascular endo-thelial growth factor (VEGF), demonstrating that aloe-emodin targets multiple molecules necessary for tumorigenesis. Cell cycle arrest in WiDr cells occurred in the G2/M phase with inhibition of cyclin B1. Another study showed that apoptosis was induced through caspases-6/9, with specific caspase-6 activation by aloe-emodin [11].

## 3.4. Gastric cancer

Gastric carcinoma (AGS, NCI-N87) cells treated with aloe-emodin demonstrated mitochondrial release of apoptosis-inducing factor (AIF) and cytochrome c-mediated activation of caspase-3[12]. AGS cells showed greater sensitivity to al-oe-emodin than NCI-N87 cells. Another study showed that MKN45 cell growth was substantially inhibited by both aloe-emodin and emodin, but more so by emodin [13]. These cells were arrested in the G0/G1 and G2/M phase by aloe-emodin and emodin, respectively. Time- and dose-dependent inhibition was demonstrated in MGC-803 cells, with an increase in S phase and a decrease in ALP activity [8]. Another study showed a cytostatic effect in MGC-803 and SGC-7901 cells, with a significant decrease in cell migration [14]. SGC-7901 cells became arrested in the G2/M phase in a time and dose-dependent manner, with a decrease in cell cycle-inducing proteins.

## 3.5. Leukemia

Monoblastic leukemia (U937) cells were treated with aloe¬emodin, resulting in reduced proliferation rate. Reactive oxygen species (ROS) and NO production, phagocytosis, and intracel-lular acidity also increased [15], the significance of which is currently elusive.

## 3.6. Lung cancer

Researchers in one study on human lung non-small cell car¬cinoma (H460) treated the cells with aloe-emodin and exam¬ined the cells with 2D electrophoresis. They found a time- de¬pendent reduction in ATP, lower ATP synthase expression, and increased mitochondrial damage with unaffected lactate dehy-drogenase (LDH) levels, suggesting the induction of apoptosis. HSP60, HSP70, and protein disulfide isomerase increased, which were hypothesized to cause apoptosis by augmenting endoplasmic reticulum stress [16].

Another series of five different studies by Lee et al. evalu-ated aloe-emodin and emodin in lung squamous carcinoma (CH27) and lung non-small cell carcinoma (H460). The first study demonstrated apoptotic changes through nuclear mor-phological change, DNA fragmentation, increased the relative abundance of cytochrome c levels, activation of caspase-3, and decreased levels of PKC isozymes generally [17]. The second study showed that CH27 cells underwent apoptotic cell death in an irreversible dose- and time-dependent manner, which coin¬cided with DNA fragmentation. BAK, BAX, and cytochrome c were elevated in the cytosol, consistent with the intrinsic apoptosis pathway [18]. In the third study, aloe-emodin treat¬ment was associated with an increased release of nucleophos-min into the cytoplasm, but no change in its gene expression [19]. Nucleophosmin is a nucleolar phosphoprotein that hyp-eraccumulates in the nucleoplasm of malignant cells and de¬creases with drug-induced apoptosis. This study showed that nucleophosmin protein levels were increased, but that the pro¬tein predominantly localized to the cytoplasm in its (inactive) proform. It was concluded that this could be a possible mecha¬nism in aloe-emodin-induced apoptosis in cancer cells. In the fourth study, aloe-emodin caused single strand DNA breaks and a decrease in the levels of DNA repair enzymes [20]. The final study supported programmed cell death via anoikis and apoptosis of H460 cells through photo-activated aloe-emodin [21]. Anoikis is a form of programmed cell death whereby the cell separates from its environment and eventually dies be¬cause it no longer receives nutrients from its surroundings. In apoptosis, specific cell signals are given to the intact cell to shut down. Increased protein expression of a-actinin and mi-togen-activated protein (MAP) kinase members was observed, and apoptosis was mediated through caspase-dependent intrin¬sic and extrinsic pathways.

In another study, aloe-emodin treatment resulted in time-and dose-dependent irreversible cell death of human lung non-small cell carcinoma (H460) [22]. Aloe-emodin decreased BCL-2, which abrogated the inhibition of pro-apoptotic pro-teins (such as BAK and BAX) and increased gene expression of p38 and caspase-3 activity, exacerbating apoptosis.

## 3.7. Liver cancer

Aloe-emodin inhibited cell growth and induced apoptosis in hepatoma (Huh-7) cells in a time- and dose-dependent manner [23]. DNA fragmentation and ROS levels were increased with a reduction in CAPN2 (calpain-2) and UBE3A (ubiquitin pro¬tein ligase E3A). These two proteins are involved in protein degradation via proteasomal processing, which enables maintenance of normal cellular activity. With their decrease, cells are unable to function and undergo apoptosis. Another study showed that aloe-emodin-treated hepatocellular carci¬noma (HepG2) cells underwent apoptosis through a cas-pase-dependent pathway with cleavage and activation of caspases-3/9 and cleavage of PARP [24]. Execution of intrinsic apoptosis was supported by translocation of cytochrome c. Hepatocellular carcinoma (HCCM and Hep3B) cells under¬went apoptosis via caspases-3/9 and PARP. Activation of p38 was unaffected in all three cell lines. Aloe-emodin-induced apoptosis was seen through oxidative stress and sustained c-JUN N-terminal kinase (JNK) activation.

## 3.8. Nasopharyngea cancer

Two studies by Lin et al. showed the effects of aloe-emodin in nasopharyngeal carcinoma (NPC) cells. The first study de¬monstrated that aloe-emodin induced apoptosis via caspase-3 activation with DNA fragmentation [25]. Cell cycle arrest in the G2/M phase was associated with increased cyclin B1-CDC2 complex formation. Matrix metalloproteinase-2 was significantly decreased, with an increase in ROS and cytosolic calcium. The second study showed that aloe-emodin signifi¬cantly inhibited cell growth without affecting viability [26]. Cyclin B1 binding to CDK1 was induced, and aloe-emodin triggered cell cycle arrest in the S and G2/M phase.

## 3.9. Neuroectodermal cancer

A study demonstrated dose-dependent cytotoxicity of aloe-emodin in neuroblastoma cells (SJ-N-KP wild-type p53 and SK-N-Be(2c) mutant p53 type) [27]. The SK-N-Be(2c) cells lack transcriptional activity of p53-targeted genes, which al-lowed studying the effect of aloe-emodin in terms of apoptosis. Expression of p53 mRNA was increased in both cell lines, but only SJ-N-KP cells showed an increase in p21, BCL-2, BAX, and CD95 mRNA due to loss of p53 function in SK- N-Be(2c) cells. Both cell lines had a time-dependent increase in p53 levels, with induction of p21 and CD95 protein expression in SJ-N-KP cells.

In addressing glial tumors, one group of researchers treated a transformed glia cell line (SVG) and a glioma U-373MG cell line with aloe-emodin, which delayed the number of cells en-tering and exiting the S phase, indicating inhibited S phase progression [28]. Another study showed aloe-emodin-induced apoptosis of U87 malignant glioma cells through disruption of mitochondrial membrane potential, cell cycle arrest in the S phase, and DNA fragmentation in a time-dependent manner with minimal necrosis [29].

## 3.10. Oral cancer

A time- and dose-dependent inhibition of cell growth was found in oral cancer (KB) cells treated with aloe-emodin, with cell cycle stalling in the G2/M phase and a decrease in S phase [30]. ALP activity was increased and no DNA fragmentation was observed.

## 3.11. Ovarian cancer

HO-8910M ovarian carcinoma cells were evaluated for mi¬gration and invasion [31]. Migration, invasion, and adhesion were significantly inhibited by aloe-emodin, with a corre¬sponding decrease in focal adhesion kinase (FAK; involved in cellular mobility, and in this case, metastasis) protein expres¬sion and mRNA levels. Aloe-emodin use in these cells attested to its anti-metastatic potential.

## 3.12. Prostate cancer

Tumor growth suppression was noted in prostate cancer (PC3) cells treated with aloe-emodin. The normal growth of prostate cells is through mTORC2 and its downstream effects. Following treatment with aloe-emodin, mTORC2's down¬stream enzymes, AKT and PKCa, were inhibited and hence exhibited decreased phosphorylation activity in a dose-depe¬ndent manner [32]. Aloe-emodin did not affect MAPK, p38, or c-JNK or phosphorylation of ERKs. Proliferation of PC3 cells was inhibited as a result of aloe-emodin binding to mTORC2, with inhibition of mTORC2 kinase activity.

## 3.13. Skin cancer

Aloe-emodin had a greater cytotoxic effect in non-mela¬noma cancer cells (epidermoid carcinoma (A431) cells and head and neck squamous cell carcinoma (SCC25) cells) than non-cancerous skin cells (pre-malignant keratinocytic HaCaT cells and Hs68 fibroblasts) [33]. This occurred through upreg-ulation of tumor necrosis factor-a (TNF-a), FAS ligand, and the associated death domains for TNF-R1 and FAS. Aloe-e-modin activated caspases-3/7/8/9 and upregulated p53, cyto-chrome c, and BAX. Intracellular ROS increased, while intra-cellular-reduced glutathione (GSH) was depleted and BCL-2 (anti-apoptotic protein) was down-regulated. Further, al-oe-emodin inhibited cytosolic *N*-acetyltransferase 1 (NAT1) enzyme activity and mRNA expression in A375.S2 malignant melanoma cells in a dose-dependent manner [34]. NAT1, ex-pressed in many human cancer cell lines, is an enzyme that N-acetylates arylamine carcinogens and drugs (initial metabo-lism) in A375.S2 cells as well as other cancer cell lines.

Aloe-emodin also sensitizes skin cancer cells to chemo-therapy. A combination of aloe-emodin and 5-fluorouracil caused an increase in cell death, as did liposomally delivered aloe-emodin. Another study showed that aloe-emodin and emodin potentiated the therapeutic effects of cisplatin, doxo-rubicin, 5-fluorouracil, and tyrosine kinase inhibitor STI 571 in Merkel cell carcinoma, which is known to be resistant to antineoplastic agents [35]. Aloe-emodin had a small advantage over emodin with respect to anti-proliferative effects when combined with these chemotherapeutic drugs at low concen¬trations, while aloin showed no effect.

Radovic et al. found that aloe-emodin caused A375 mela-noma cells to undergo apoptosis accompanied by BCL-2 downregulation and caspase-mediated apoptosis [36]. An in-teresting finding was that aloe-emodin rescued cells from dox-orubicin- or paclitaxel-induced death in a dose-dependent manner, exhibiting a cytoprotective effect. Accordingly, cau-tion is warranted when using aloe-emodin with these chemo-therapy drugs. Finally, aloe-emodin significantly inhibited Merkel cell carcinoma growth with no effect by aloin [37]. Basic fibroblast growth factor (bFGF), transforming growth factor-p1 (TGFp1), nerve growth factor (NGF), and epidermal growth factor (EGF) did not affect proliferation of Merkel cell carcinoma cells.

## 3.14. Tongue cancer

Chen and colleagues investigated the effects of aloe-emodin, emodin, and rhein on SCC-4 tongue squamous cell carcinoma in two studies. The first study revealed a decrease in viability in a time- and dose-dependent manner, with the greatest effect induced by emodin, followed by aloe-emodin, then rhein [38]. Migration and invasion of SCC-4 cells was inhibited, with reductions in MMP-2 and NF-KB, signifying decreased cell mobility. In the second study, cytotoxicity and induction of DNA damage were seen in the same order of magnitude per anti-carcinogenic agent [39]. Expression of DNA-PK, BRCA1, and ATM mRNA (all DNA repair enzymes) was significantly inhibited by aloe-emodin, with varying effects by emodin and rhein. Another study showed that aloe-emodin inhibited SCC-4 cell viability in a dose-dependent manner with S phase arrest and changes in nuclear morphology [40]. Levels of ROS, cal¬cium, and caspases-3/8/9 activity increased in a time-de¬pendent manner, accompanied by a reduction in mitochondrial membrane potential.

## 3.15. General/other

Aloe-emodin demonstrated p53-independent apoptosis in FaDu (pharyngeal squamous cell carcinoma), Hep3B (hepa¬toma), and MG-63 (osteosarcoma) cells [41]. This resulted in S phase cell cycle arrest. Caspase-8/10-associated RING pro¬tein (CARP) expression was decreased by aloe-emodin. When CARPs were overexpressed, aloe-emodin-induced apoptosis was attenuated. CARPs normally interact with caspase-8/10 by inhibiting their function through ubiquitin-mediated proteoly-sis. With decreased levels of CARPs, apoptosis is increased.

**Table 1.**
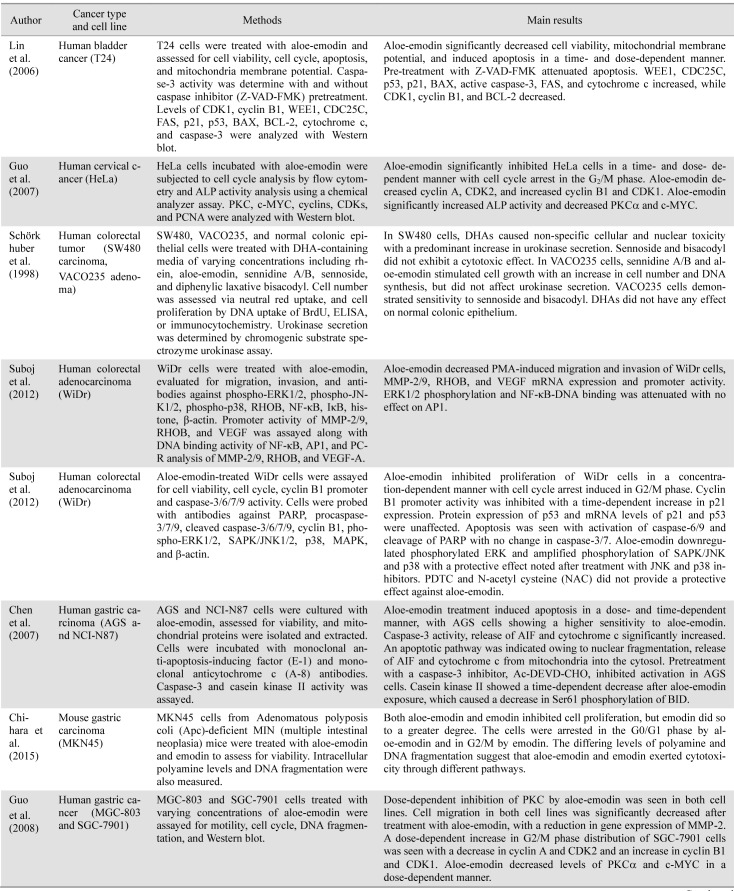
Summary of biochemical effects of aloe-emodin and similar anthraquinones in human-derived cancer cell lines

## 4. Discussion

This review of *in vitro*data clearly suggests that aloe-emodin, an inexpensive, readily available nutrient from aloe vera with a longstanding history of safe use, has an-ti-neoplastic and anti-proliferative effects on multiple cancer types and cell lines. A secondary finding from selected studies suggests that aloe-emodin has great potential to serve as an adjunct to conventional chemotherapeutic regimens, as these data demonstrate potential for synergy with selected chemo-therapeutic agents, allowing for a reduction in drug dose. Nev¬ertheless, one study showed cytoprotective effect of al-oe-emodin on cancer cells, possibly leading to a restricted ef¬ficacy of selected chemotherapeutic agents.

Aloe-emodin clearly induced cancer cell apoptosis in multi-ple studies, involving various types of cancer and different cell lines. Specifically, aloe-emodin's anti-cancer properties *in vitro*emanate mainly from cell death induction and an-ti-proliferative processes that entail (a) increased levels of pro-apoptotic caspases, cytochrome c, p53 and p21, BAX, and free radicals, and decreased levels of anti-apoptotic BCL-2 and DNA repair enzymes; (b) decreased cyclin A, CDK2, VEGF, and NF-KP levels; (c) cell cycle arrest in the S and G2/M phase; and (d) decreased MMP levels and migration. A key *in vitro*finding was the increased release of cytochrome c, the molec¬ular initiator of intrinsic apoptosis in many cancer cell lines, including: non-small cell lung carcinoma (H460) [16,17], squamous cell lung carcinoma (CH27) [18], hepatocellular carcinoma (HepG2, HCCM, and Hep3B) [24], nasopharyngeal carcinoma (NPC) [25,26], human neuroblastoma (SK-N-Be(2c) and SJ-N-KP) [27], premalignant keratinocytic (HaCaT) cells [33], skin fibroblast (Hs68) [33], epidermoid carcinoma (A431) [33], head and neck SCC (SCC25) [33], skin melanoma (A375) [33,34,36], and tongue squamous cell carcinoma (SCC-4) [40].

When co-administered with a variety of anti-neoplastic agents, aloe-emodin served as a chemosensitizer. In Merkel cell carcinoma, aloe-emodin exhibited synergistic effects with cisplatin, doxorubicin, 5-fluorouracil, and the tyrosine kinase inhibitor STI 571 [35]. Conversely, aloe-emodin demonstrated increased protection in malignant melanoma cells against doxorubicin and paclitaxel (A375) [36]. The authors proposed that aloe-emodin protects cells once they have been exposed to toxic molecules and not necessarily against chemotherapy.

Furthermore, the pharmacological efficacy of liposomal aloe-emodin was more profound versus non-encapsulated aloe-emodin, indicating that aloe-emodin may be formulated into nanoparticulate drug delivery systems to increase the dis-tribution of the active ingredient. Increased ROS and cas-pase-3/7/8/9 with decreased GSH were noted in this study [33]. This particular study also demonstrated similar anti-tumor pro¬perties in additional cell lines, including premalignant keratinocytic (HaCaT), skin fibroblast (Hs68), epidermoid carcinoma (A431), head and neck SCC (SCC25), and skin melanoma (A375) cells, attesting to aloe-emodin's efficacy against a wide range of target cell types.

Additional studies are needed to gather clinical knowledge and to investigate the potential use of aloe-emodin and related compounds as adjuvants in conventional cancer treatment. For example, a study by Lissoni et al. showed that *Aloe ar-borescens*combined with chemotherapy improved solid tumor regression and survival time in patients with lung, colorectal, gastric, and pancreatic cancer [42]. Insofar as aloe-emodin at higher doses was shown to inhibit chemotherapy in one iso¬lated study, it is particularly important to further investigate the potential for drug-supplement interactions.

Several limitations of this review are noteworthy. With this area of research on aloe-emodin being fairly recent, dysregula-tion at the transcript and protein level was found, but the mechanisms are poorly understood. This review only focuses on *in vitro*studies, which could prove to have limited trans-latability to *in vivo*studies. Additionally, multiple studies not¬ed the fact that aloe-emodin could possibly be used prophylac-tically. As far as we are aware, no studies have yet been un¬dertaken to test this hypothesis. Finally, few studies to date have investigated the effects of aloe-emodin in noncancerous cell lines. Such studies are warranted to determine whether aloe-emodin exerts similar cytotoxic effects in typically slow-proliferating and quiescent cells. Cytotoxicity in cancer cells and no effects in non-malignant cells would be the de¬sired outcome and a rudimentary gauge of a compound's target specificity (i.e., highly proliferative cancer cells).

In summary, aloe-emodin shows great promise as an an-ti-neoplastic agent with potential use as a synergistic and/or cytoprotective agent as part of conventional cancer treatment. Numerous *in vitro*results support this claim, yet further re¬search is needed to elucidate the molecular mechanisms *in vivo,*as well as to investigate its potential use as a prophylactic agent clinically. Aloe-emodin may ultimately prove to be another phytonutrient with anti-cancer properties.

## List of abbreviations:

14-3-3a: regulatory protein (also known as stratifin) expressed in all eukaryotic cells, helps with translocation to cell membrane as well as signal transduction; 2-DE: 2-dimensional gel electrophoresis, used to sort out different proteins based on their size, charge, and other biochemical properties; Ac-DEVD-CHO: specific, potent, and reversible inhibitor of caspase-3; AIF: apoptosis-inducing factor; AKT: also known as protein kinase B, inhibits apoptosis; ALP: alkaline phosphatase, dephosphorylates proteins helping to break cells down; AP1: acti¬vator protein-1, a transcription factor; ATF-2: activating transcription factor-2; ATM: ataxia telangiectasia mutated; phosphorylates key regulatory proteins leading to cell cycle arrest, DNA repair, or apoptosis; ATP: adenosine triphosphate, molecule used as energy for the cell to carry out many functions in the body; ATR: ataxia telangiectasia and Rad3-related protein, checks for DNA damage and initiates repair; BAD: BCL-2-associated death promoter protein, helps to initiate apoptosis; BAG-1: BCL-2-associated anthanogene, binds to Bcl-2 and helps to prevent apoptosis; BAK: BCL-2 homologous antagonist/killer; BAX: BCL-2-associated X protein, induces apoptosis; BCL-2: B-cell lymphoma 2, inhib¬its apoptosis by preventing release of cytochrome c, has many different forms and can also induce apoptosis; BCL-XL: B-cell lymphoma extra-large, inhibits apoptosis by preventing the release of cytochrome c; bFGF: basic fibroblast growth factor, induces cell proliferation; BID: BH3-interacting domain death agonist, works in conjunction with BAX to become pro-apoptotic; BIK: BCL-2-interacting killer, destroys BCL-2 to induce apoptosis; BIM: BCL-2-like protein 11, part of the BCL-2 family but this protein induces apoptosis; BRCA1: breast cancer 1, a tumor suppressor gene that, if mutated, can be the cause of hereditary breast cancers; BrdU: 5-bromo-2'-desoxyuridine, a thymidine analogue used to detect cell proliferation; cAMP: cyclic adenosine monophosphate, derivative of ATP, second messenger involved in cell proliferation; CAPN2: calcium-activated neutral proteases, breaks down protein; CARP1/2: caspase-8/10-associated RING protein, inhibit caspase 8 and 10, decreasing apoptosis; CD11b/14/95: cluster of differentiation, integrin expressed on the surface of leukocytes; CDC25C: cell division cycle 25c, triggers entry into mitosis; CDK1/2: cyclin-dependent kinase 1/2, once phosphorylated it progresses the cell cycle; CDT1: chromatin licensing and DNA replication factor 1, 'licenses' DNA to flag it ready for replication; CKI: cyclin-dependent kinase inhibitors; c-MYC: Myc proto-oncogene; has a role in cell cycle progression, apoptosis, and cell transformation; CsA: cyclosporin A, caspase 8 inhibitor; Cu: copper; DAPI: 4',6-diamidino-2-phenylindole, fluorogenic probe with an affinity for A-T-rich regions of DNA; DHA: dihydroxyanthraquinone, class of mole¬cules to which aloe emodin and structurally related compounds belongs to; DMSO: dimethylsulfoxide, polar aprotic solvent; DNA: deoxyribonu-cleic acid; DNA-PK: deoxyribonucleic acid-dependent protein kinase, functions during DNA repair; ELISA: enzyme-linked immunosorbent assay, used for the determination of specific proteins; ENDO G: endonuclease G, participates in caspase-independent apoptosis; ERK: extracel¬lular signal-related kinase, part of the MAP kinase pathway, functions to induce cellular proliferation; FADD: FAS-associated protein with death domain, helps to induce formation of the death complex to carry out apoptosis; FAK: focal adhesion kinase, also known as protein tyrosine kinase 2, involved in cell adhesion and spreading; FAS: Fas cell surface death receptor, mediator of extrinsic apoptosis; G2/M: G2 phase and M phase of the cell cycle, mediates growth and mitosis/meiosis, respectively; G-CSF: granulocyte colony-stimulating factor, stimulates growth of granulocytes (white blood cells); GM-CSF: granulocyte macrophage colony-stimulating factor; GRP78: 78-kDa glucose-regulated protein, also known as binding immunoglobulin protein, chaperone protein that helps newly synthesized proteins to be formed and shaped; GSH: reduced glu-tathione, an endogenous antioxidant; GSSG:glutathione disulfide (oxidized form of GSH); HOGG1: human 8-oxoguanine DNA N-glycosylase 1, a DNA repair enzyme; HSP:heat shock protein, increased in cancer cells and stressed cells, necessary for cancer cell survival; IFN: interferon, a class of molecules used in immunity to confer specific immune signals; IgG: immunoglobulin G, a type of antibody in long-term immunity; IL: interleukin, class of molecules used in immunity to confer specific immune signals; IP-10: interferon gamma-induced protein 10; IKB: inhibitor of kappa B, inhibits NF-KB signal transduction; JNK:c-JUN N-terminal kinase, helps activate pro-apoptotic proteins; JNKK1: Jun N-terminal kinase kinase; MAPK: mitogen-activated protein kinase; MCC: Merkel cell carcinoma; MCL-1: myeloid cell leukemia 1, alternative splicing occurs on this gene, one splice variant induces apoptosis while the other inhibits it; MCM complex; minichromosome maintenance protein complex, hel-icase protein required for DNA replication; MCP-1: monocyte chemotactic protein-1; MGMT: O-6-methylguanine-DNA methyltransferase, DNA repair enzyme; MIP: macrophage inflammatory protein; MMP: matrix metallo-protease, degrades extracellular matrix, facilitates metastasis; Mn: manganese; mRNA: messenger ribonucleic acid: mTOR: mammalian target of rapamycin, protein involved in cell proliferation: mTORC2: mTOR complex 2; NAT: N-acetyl transferase, initiates metabolism; NF-KB: nuclear factor kappa B, induces transcription of genes involved in many processes including inflammation, angiogenesis, and cell survival; NO: nitric oxide, causes vasodilation; NOXA: phor-bol-12-myristate-13-acetate-induced protein 1, pro-apoptotic member of the BCL-2 family; ORC: origin recognition complex; p21: cy-clin-dependent kinase inhibitor 1; p38: class of mitogen-activated protein kinases involved in cell differentiation, apoptosis, and autophagy; p53: 53 kDa protein, conserves DNA stability as a tumor suppressor; p65: REL-associated protein involved in NF-KB function; PARP: poly adenosine diphosphate-ribose polymerase, DNA repair enzyme; PCNA: proliferating cell nuclear antigen; PCR: polymerase chain reaction, used to replicate DNA sequences; PDGFbb: platelet-derived growth factor bb; PDI: protein disulfide isomerase, helps with protein folding; p-ERK: phosphory-lated (activated) extracellular signal-related kinase; PI: propidium Iodide, florescent stain used to determine cell viability; PI3-K: phosphoinosi-tide-3 kinase, enzyme involved in cell proliferation; PKA: protein kinase A, intracellular enzyme used in many pathways to sustain cell viability; PKAc: protein kinase A, catalytic subunit; PKC: protein kinase C, involved in cell proliferation; p-PERK:phosphorylated (activated) protein ki-nase RNA-like endoplasmic reticulum kinase, monitors cell homeostasis; PRDX: peroxiredoxin, a redox regulator; PS: phosphatidylserine, a phospholipid used as a signal for macrophages to engulf the cell during apoptosis; PUMA: P53 upregulated modulator of apoptosis, pro-apoptotic signaling protein; RAF:rapidly accelerating fibrosarcoma, protein kinase related to retroviral oncogenes, part of signal cascade aiding in cell pro¬liferation; RANTES: regulated on activation, normal T cell expressed and secreted , also known as chemokine (C-C motif) ligand 5 (CCL5); RAS:rat sarcoma, protein involved in signal transduction; RHOA: Ras homolog gene family member A, used in actin organization to control cell morphology; RHOB: Ras homolog gene family member B, similar to RHOA; ROCK-1: Rho-associated, coiled coil-containing protein kinase 1, helps with cell morphology and migration; ROS: reactive oxygen species; RT-PCR: reverse transcription polymerase chain reaction, to form DNA from RNA and then replicate it; SAPK: stress-activated protein kinase, homologues to JNKs; SCC: squamous cell carcinoma; siRNA: small in¬terfering ribonucleic acid, blocks protein formation (translation); SOD: superoxide dismutase, removes oxygen free radicals; SPM: spermine, involved in cell metabolism; tBID: truncated BH3-interacting death domain agonist; Thr14/Tyr15: threonine 14/tyrosine 15, ; phosphorylation of these proteins by WEE1 inhibits mitosis; TIMP-1: tissue inhibitor of metalloproteinases 1; TNF: tumor necrosis factor, cytokine causing cell death; UBE3A: ubiquitin protein ligase E3A, assists with ubiquitination or cell death; U-PA: urokinase plasminogen activator, involved in extra¬cellular matrix degradation; VEGF: vascular endothelial growth factor; WEE1: nuclear kinase, inhibits CDK1 and the cell cycle; Z-IETD-FMK: caspase-8 inhibitor, inhibits T cell proliferation and prevents oxyhemoglobin-induced cell death; Zn:zinc; Z-VAD-FMK: car-bobenzoxy-valyl-alanyl-aspartyl-[O-methyl]-fluoromethylketone, binds to the protease site of caspases and can inhibits apoptosis.
